# Site-Specific Identification of an Aβ Fibril–Heparin Interaction Site by Using Solid-State NMR Spectroscopy**

**DOI:** 10.1002/anie.201204459

**Published:** 2012-11-14

**Authors:** Jillian Madine, Maya J Pandya, Matthew R Hicks, Alison Rodger, Edwin A Yates, Sheena E Radford, David A Middleton

**Affiliations:** Institute of Integrative Biology, University of LiverpoolCrown Street, Liverpool L69 7ZB (UK) E-mail: middleda@liverpool.ac.uk; Astbury Centre for Structural Molecular Biology and Institute of Molecular and Cellular Biology, University of LeedsLeeds, LS2 9JT (UK); Department of Chemistry, University of WarwickWarwick, CV4 7AL (UK)

**Keywords:** Alzheimer’s disease, amyloid β-peptides, heparin, linear dichroism, NMR spectroscopy

Over 30 proteins and peptides self-assemble into amyloid fibrils and plaques that are the pathological hallmark of human disorders; the most famous peptides are the 40- and 42-residue amyloid beta peptides Aβ_1-40_ and Aβ_1-42_ associated with Alzheimer’s disease (AD).^1^ As early as 1850 it was suggested that amyloid deposits in tissue are associated with carbohydrates, later identified and classified as proteoglycans^2^ and glycosaminoglycan (GAG) polysaccharides,^3^ and these factors are now known to accumulate in AD plaques.^4^ GAGs, such as heparan sulfate and the closely-related polysaccharide heparin, increase the rate of Aβ polymerization^5^ and markedly affect the resulting fibril morphology^6^ and neurotoxicity.^7^ GAG mimics, which accelerate fibril deposition and eliminate cytotoxic amyloid species, are being evaluated clinically as AD therapies.^8^ Precisely how GAGs interact with Aβ fibrils is not known, but the sequence H_13_HQK is a predicted heparin binding motif^9^ and solid-state NMR (SSNMR) data suggest that the N-terminal arginine R5 is also involved.^6^ Here, we examine in detail the GAG binding interface in Aβ_1-40_ fibrils using ^13^C SSNMR spectroscopy, linear dichroism (LD), and enzymatic assays. By using 5 kDa heparin as an established proxy for GAGs,^10^ we report the first atomic details of a GAG binding site on the surface of Aβ_1-40_ fibrils based on direct structural measurements.

Aβ_1-40_ fibril morphology is highly dependent on assembly conditions,^11^ and restraints from SSNMR experiments revealed that this polymorphism originates from differences in structure and organization at the molecular level.^12^ Aβ_1-40_ fibrils assembled under quiescent conditions exhibit threefold molecular symmetry (“3Q” morphology),12b while fibrils formed with agitation exhibit twofold molecular symmetry (“2A” morphology; Figure 1 A).12a, c Here, uniformly ^13^C/^15^N-labeled Aβ_1-40_ fibril samples were prepared by elongation of 2A or 3Q fibril seeds.12b Transmission electron microscopy (TEM) confirmed that fibrils had formed successfully in these seeded growth experiments (Figure 1 C, left, and Figure S1 in the Supporting Information). Two-dimensional ^13^C–^13^C SSNMR spectra of these samples (Figure S2 in the SI) exhibited well-resolved signals consistent with the expected homogeneous fibril morphologies. These morphologies were confirmed as 2A and 3Q by comparing the diagnostic chemical shifts with documented values (Tables S1 and S2, and Figure S2 in the Supporting Information). Seeded 2A and 3Q fibrils that assembled in the presence of a fivefold mass excess of 5 kDa heparin appeared to be unaffected morphologically according to TEM (Figure 1 C). However, spectra of the two fibril types in the presence of heparin collected using the differential absorbance technique, flow LD^13^ (see the Supporting Information), were strikingly different from the spectra of the fibrils alone (Figure 1 B). Spectra of the fibrils without heparin showed a small amide signal at about 200 nm and only weak signals in the aromatic region around 280 nm (c.f. no aromatic signal was observed in Aβ_1-42_ fibrils in reference [12]). This result indicates that fibrils are present, but they do not orient particularly well in shear flow. The 3Q fibrils give the higher LD signal, possibly because the characteristic higher mass-per-length of 3Q fibrils^11^ results in stiffer fibrils, which align better than 2A fibrils. For fibrils assembled in the presence of heparin, a larger LD signal is observed in the amide region for both 2A and 3Q fibrils, but the magnitude of the signal enhancement is different for the two morphologies (Figure 1 B). These data indicate that heparin binds to both fibril types, and the observed enhancement in LD signal results either from a difference in their binding affinity for heparin, and/or from differences in the ability to align the heparin-bound fibrils under flow. Stronger signals observed at 280 nm and 240 nm (Figure 1 B, insets) are consistent with alterations in tyrosine–tyrosine interactions involving the sole tyrosine (Y10) near the flexible N terminus.^14^ Furthermore, small peaks between 250 nm and 280 nm that are due to ordering of the phenylalanines (F4, F19, and/or F20)^14^ are observed. To quantify heparin binding directly, an experiment was designed in which fibrils in the presence of heparin were sedimented and the heparin concentration in the supernatant was determined enzymatically using heparinase I. Heparin binding to 2A fibrils (Figure 1 D, •) is considerably weaker than binding to 3Q fibrils, the latter of which reached saturation at sub-equimolar concentrations (Figure 1 D, ▪). Hill analysis of the data for 3Q suggests that heparin binding is cooperative with an estimated *K*_D_ of 34 μm and a stoichiometry of approximately one heparin molecule per four Aβ_1-40_ molecules at saturation (i.e., 4.5 saccharide units per Aβ_1-40_ monomer). The LD and enzymatic experiments together reveal marked differences in heparin binding to the two Aβ_1-40_ morphologies.

**Figure 1:**
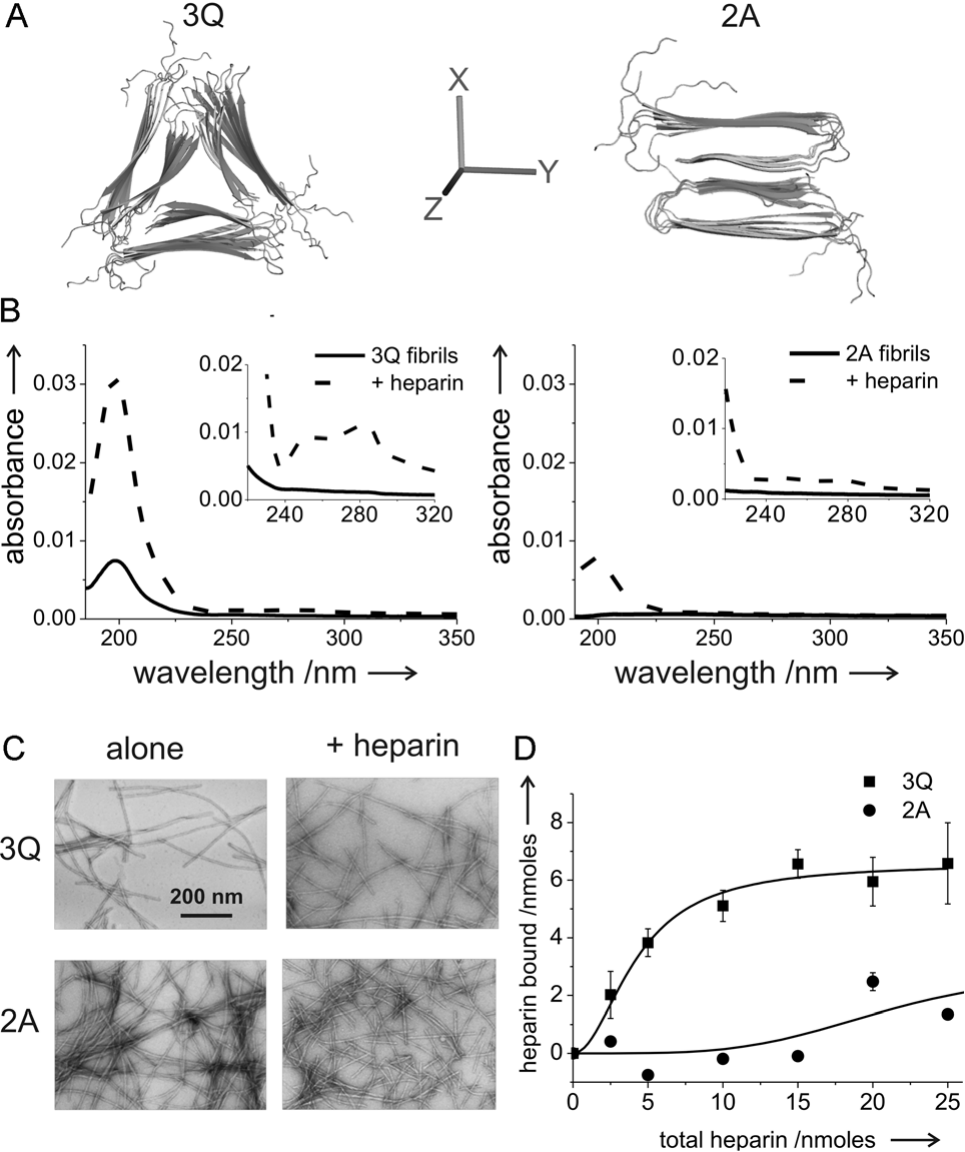
Binding of heparin to fibrils of Aβ_1-40_ with different morphology. A) Models of the 3Q (left) and 2A (right) fibrils viewed down the fibril long axis Z.^9^ Each model comprises six layers of hydrogen-bonded peptides produced using coordinate pdb files, from Dr. Robert Tycko, with flexible N-terminal residues (not restrained by NMR data) added. B) Flow LD spectroscopy of 3Q (left) and 2A (right) fibrils (0.9 mg mL^−1^) in the absence or presence of a fivefold mass excess of 5 kDa heparin. Samples were diluted 20-fold to reduce absorbance at lower wavelengths. Insets show long-wavelength LD spectra of undiluted fibrils in the absence or presence of a fivefold mass excess of 5 kDa heparin. C) TEM images of 3Q (top) and 2A fibrils (bottom). Fibrils were grown alone or with a fivefold mass excess of 5 kDa heparin added during fibril formation. Scale bar is 200 nm in all images. D) Heparin binding to 3Q (▪) and 2A fibrils (•). Results shown as mean ± standard error of the mean (S.E.) from experiments carried out in triplicate. Data were analyzed using nonlinear regression fitted using the Hill equation (solid lines). The fibrils were suspended at 0.9 mg mL^−1^ before sedimentation.

Further 2D ^13^C–^13^C SSNMR spectra were obtained for 2A and 3Q fibrils prepared by seeding in the presence of a fivefold mass excess of heparin. Chemical shift perturbations for selective main-chain carbon atoms or side-chain carbon atoms were indicated by significant movements of some cross-peaks for 3Q fibrils in the presence of heparin (Figure 2, left panels, and Figure S4 A in the Supporting Information). Similar perturbations were observed when heparin was added after preassembly of the 3Q fibrils (Figure S5 in the Supporting Information), thereby suggesting that the interaction site is situated on the external face of the fibril assembly. Residues significantly perturbed in the presence of heparin are E22, S26, N27, A30, I31/I32, and H13/H14, with additional perturbations of the visible cross-peaks for residues in the flexible N terminus (E3, R5, and H6) (Figure 2 B, left and Figure S6 in the Supporting Information). Furthermore, cross-peaks for residue D23 are visible only in the spectrum obtained in the presence of heparin (Figure 2 A, left, red). Chemical shift perturbations observed in the aromatic region are consistent with the aromatic interactions detected in the LD experiments, but spectral overlap prevented the identification of specific residues. Figure 3 A highlights the positions of the heparin-sensitive residues on the modeled structure of 3Q fibrils and reveals that they are clustered in the unstructured N-terminal region (residues 1–8) and the turn at the apices of the triangular geometry. In contrast, very few significant changes in chemical shifts were observed for the 2A fibrils in the presence of heparin (Figure 2 B, right panels, and Figure S4B in the Supporting Information), consistent with lower-affinity heparin binding to this morphology.

**Figure 2:**
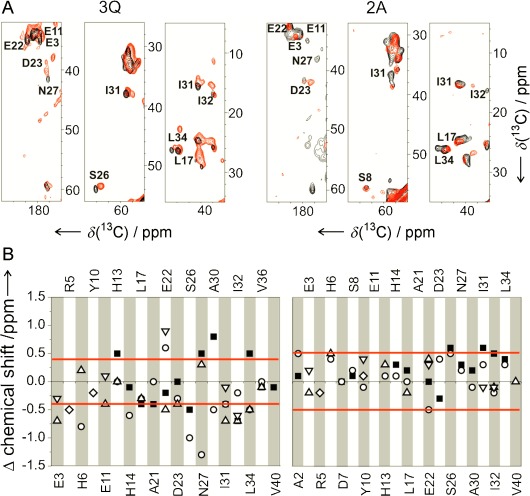
^13^C dipolar assisted rotational resonance (DARR) SSNMR spectra of Aβ_1-40_ fibrils. A) Regions for 3Q (left) and 2A (right) fibrils alone (black) and incubated in the presence of heparin (red). Full spectra are shown in Figure S4 in the Supporting Information. B) Heparin-induced chemical shift perturbations for 3Q (left) and 2A (right) fibrils. The regions bounded by red lines indicate the mean linewidth at half height (see Figure S6 in the Supporting Information). Significant perturbations are taken to be >|±50 %| of the mean half-height linewidths for 3Q and 2A. Some resonances (e.g., for some valine residues) could not be assigned unambiguously owing to spectral overlap. Cα=▪, Cβ=○, Cγ=▵, Cδ=▿, Cζ=◊.

**Figure 3:**
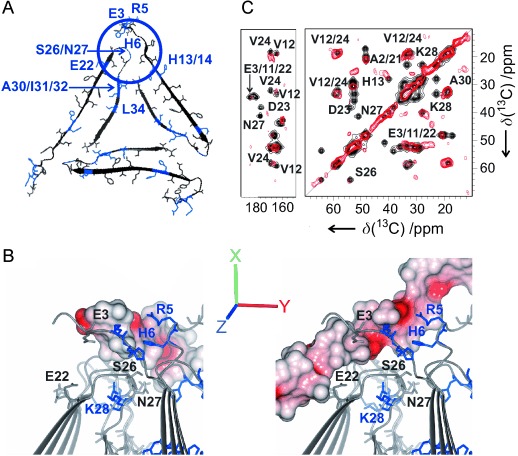
Models for the GAG–fibril interface for 3Q fibrils. A) A single threefold symmetric layer showing the proposed heparin binding region (circled). Residues that undergo significant chemical shift perturbations in the presence of heparin are highlighted in blue. B) Models of heparin bound to 3Q fibrils were generated using chemical shift perturbations to define the search area. The binding site is defined by residues in the turn (E22, S26, N27, K28) and flexible N terminus (E3, R5, H6). Side chains from residues that show chemical shift perturbations in the presence of heparin are displayed, with positively charged residues highlighted (blue). The negatively charged surface of heparin is shown in red. Alternative arrangements are shown with heparin positioned parallel with (left) and perpendicular to (right) the fibril axis Z. Images were generated using *CCP*4 *mg* molecular graphics software.^16^ C) Experimental PSD spectrum (red) and simulated spectrum (black) corresponding to residues encompassed by the circled region in (A). Details of the docking and NMR simulations are available in the Supporting Information.

Independent rounds of simulations of heparin docking with a model of 3Q fibrils comprising 18 Aβ_1-40_ molecules were carried out using a search area (Figure 3 A) encapsulating residues for which significant chemical shift perturbations were observed. No constraints were imposed on the orientation of heparin and simulations indicated that heparin can associate with the fibril surface either parallel with the fibril axis (Figure 3 B, left, and Figure S7 in the Supporting Information) or perpendicular to the fibril axis (Figure 3 B, right, and Figure S7). Favorable free energies of binding were calculated for the two heparin orientations (−5.5 kcal mol^−1^ for parallel and −2.8 kcal mol^−1^ for perpendicular). A perpendicular orientation allows the heparin/Aβ_1-40_ stoichiometry of 1:4 estimated from the heparinase data, but this stoichiometry is not possible in a parallel orientation unless there is a second interaction site, perhaps involving the H_13_HQK motif or the aromatic residues producing the enhanced LD signal. Heparin binding to the 3Q fibrils was probed further in a ^13^C-detected proton spin diffusion (PSD) NMR experiment^15^ (Figure S8 in the Supporting Information). Cross-peaks (Figure 3 C, red) arise only from residues in closest contact with the ligand and residues that are remote from the ligand remain silent. A simulated spectrum (Figure 3 C, black) for residues within the circled binding region agrees well with the experimental spectrum, with overlap of many cross-peaks, although there are differences. Some predicted cross-peaks are absent from the experimental spectrum (e.g., for D23 and N27), and additional cross-peaks are observed that are not accounted for in the simulated spectrum (e.g., for H14). These discrepancies may arise because the complex spin-diffusion pathway is not modeled adequately, and because additional heparin binding sites may contribute to the spectrum. Crucially, spectra simulated for other possible binding sites outside the proposed region fit much more poorly with the experimental spectrum (Figure S9 in the Supporting Information).

In summary, we report the first detailed molecular insights into a recognition site for heparin, which is a proxy for GAG, within Aβ_1-40_ fibrils. GAGs are ubiquitous components of amyloid deposits in vivo and may be complicit in amyloid pathogenicity.^18^ A fascinating outcome of this work is the profound difference seen in the binding of heparin to the 2A and 3Q fibril types. The distinct packing geometries of the cross-β units of the 2A and 3Q fibrils may explain the different affinity of heparin for the two morphologies. Specifically, in the 3Q fibrils the N-terminal region lies in close proximity to the loop that links the two β strands in each monomer. This arrangement is not found in the 2A fibrils, thus possibly rationalizing the unique ability of heparin to bind tightly to fibrils of the 3Q type. Importantly, we show that GAG–amyloid interactions can differ according to the morphology of fibrils assembled from the same protein sequence. Further structural studies will be needed to determine how the binding site and mechanism differs in fibrils and their polymorphs formed from other protein sequences known to colocalize with GAGs, or how binding to the fibrils is influenced by GAG length. This work establishes a platform for such studies and for the structure-inspired design of compounds that target GAG–polypeptide interactions for therapeutic or diagnostic purposes.^9^, ^19^

## Experimental Section

SSNMR experiments were performed below −20 °C on Bruker 400 MHz and 850 MHz spectrometers at MAS frequencies of up to 14 kHz. LD spectra of aligned fibrils were recorded using a microvolume Couette cell alone and in the presence of heparin. Molecular docking simulation of heparin onto Aβ_1-40_ seeded fibrils was performed using the AutoDock Vina 1.0 package.^17^ Heparin binding was determined using heparinase cleavage of heparin remaining in solution following centrifugation (15 min, 14 000 g) of Aβ_1-40_ fibrils and associated heparin. Heparinase I cleaves the glycosidic linkage, thereby giving unsaturated uronic acid, which can be detected by measuring the absorbance at 232 nm. Detailed experimental and computational methods are available in the Supporting Information. Coordinate files of the structural models are available from the authors on request.

## Supplementary material

Detailed facts of importance to specialist readers are published as ”Supporting Information”. Such documents are peer-reviewed, but not copy-edited or typeset. They are made available as submitted by the authors.
