# Supplementary material for: Site-Specific Identification of an Aβ Fibril–Heparin Interaction Site by Using Solid-State NMR Spectroscopy
Source: Angew Chem Int Ed Engl. 2012 Nov 14;51(52):13140–3. doi: 10.1002/anie.201204459 (PMC3749465; doi:10.1002/anie.201204459)
Supplement: Supplementary file 1 [file anie0051-13140-SD1.pdf]

Angewandte Chemie
Eine Zeitschrift der Gesellschaft Deutscher Chemiker

Supporting Information

© Wiley-VCH 2012

69451 Weinheim, Germany

Near-Atomic Resolution Neutron Crystallography on Perdeuterated *Pyrococcus furiosus* Rubredoxin: Implication of Hydronium Ions and Protonation State Equilibria in Redox Changes**

*M. G. Cuypers, S. A. Mason, M. P. Blakeley, E. P. Mitchell, M. Haertlein, and V. Trevor Forsyth**

anie_201207071_sm_miscellaneous_information.pdf

Supporting Information

Table of Contents

Page 2	Figure S1: Negatively stained TEM images of 3Q and 2A fibrils in the presence and absence of a 5-fold mass excess of 5 kDa heparin.
Page 3	Figure S2: Diagnostic regions of ^{13}C-^{13}C dipolar-assisted rotational resonance (DARR) SSNMR spectra of $A\beta_{1\text{-}40}$ fibrils.
Page 4	Figure S3: Assignment of ^{13}C-^{13}C DARR SSNMR spectra.
Page 5	Figure S4: Two-dimensional ^{13}C SSNMR spectra of seeded $A\beta_{1\text{-}40}$ fibrils assembled in the absence or presence of heparin.
Page 6	Figure S5: Two-dimensional ^{13}C SSNMR spectra of 3Q $A\beta_{1\text{-}40}$ fibrils formed in the presence of heparin or formed alone with heparin added post-fibril formation.
Page 7	Figure S6: Residue-specific ^{13}C NMR line widths for $C\alpha$ and $C\beta$ of $A\beta_{1\text{-}40}$ fibrils.
Page 8	Figure S7: Autodock Vina generated models of heparin bound to 3Q fibrils.
Page 9	Figure S8: Details of the ^{13}C-detected proton spin diffusion experiment.
Page 10	Figure S9: Comparison of the experimental ^{13}C-detected proton spin diffusion spectrum with simulated spectra.
Page 11	Table S1: Summary of ^{13}C chemical shifts for 3Q $A\beta_{1\text{-}40}$ fibrils.
Page 12	Table S2: Summary of ^{13}C chemical shifts for 2A $A\beta_{1\text{-}40}$ fibrils.
Page 13	Detailed Methods

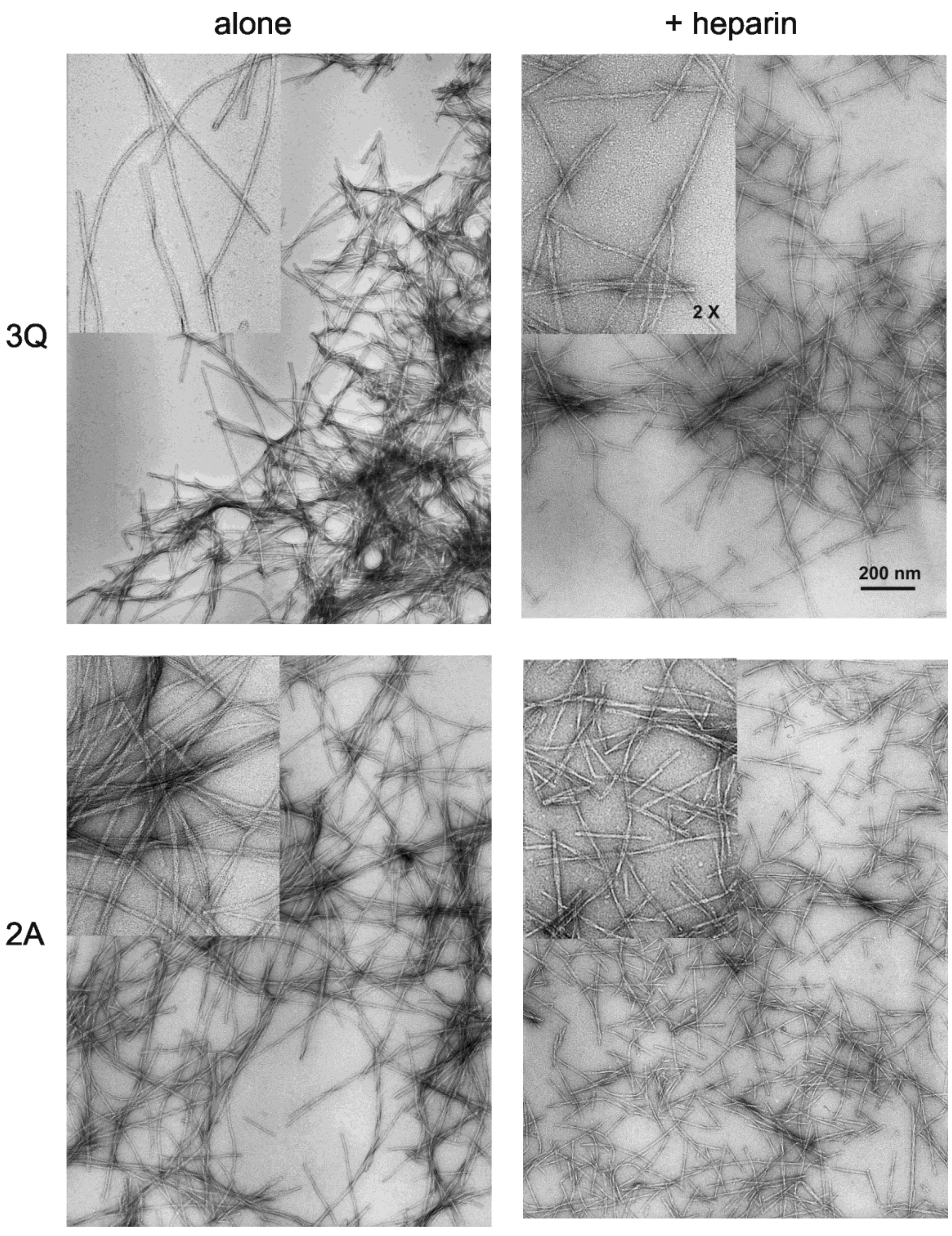

Figure S1: Negatively stained TEM images of 3Q and 2A fibrils in the presence and absence of a 5-fold mass excess of 5kDa heparin. The inset shows an expansion (2x).

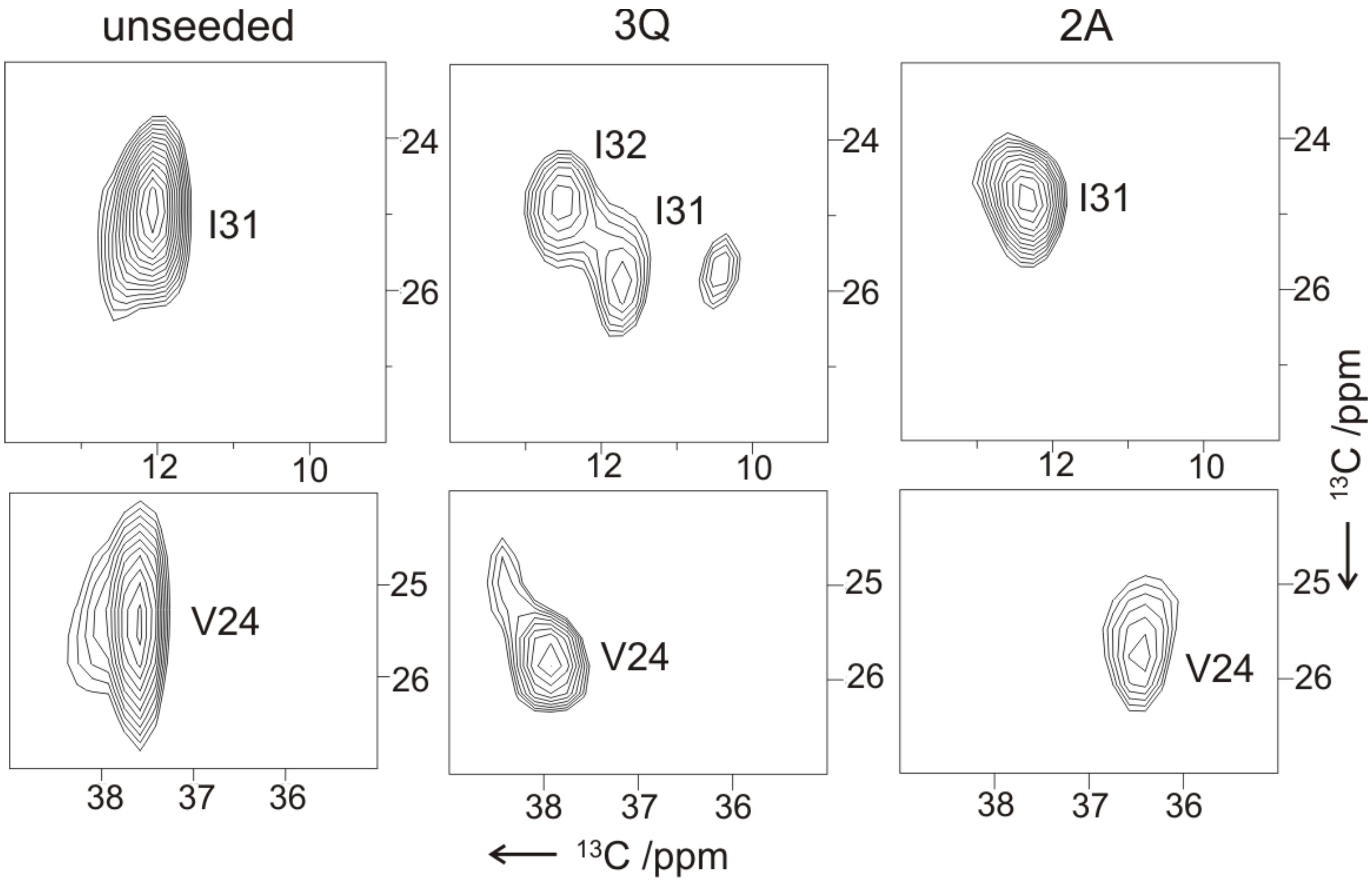

Figure S2: Examples of diagnostic regions of ^{13}C-^{13}C dipolar-assisted rotational resonance (DARR) SSNMR spectra which distinguish between unseeded fibrils of A$\beta_{1\text{-}40}$ and fibrils of the type 3Q and 2A formed by seeded elongation.[1]

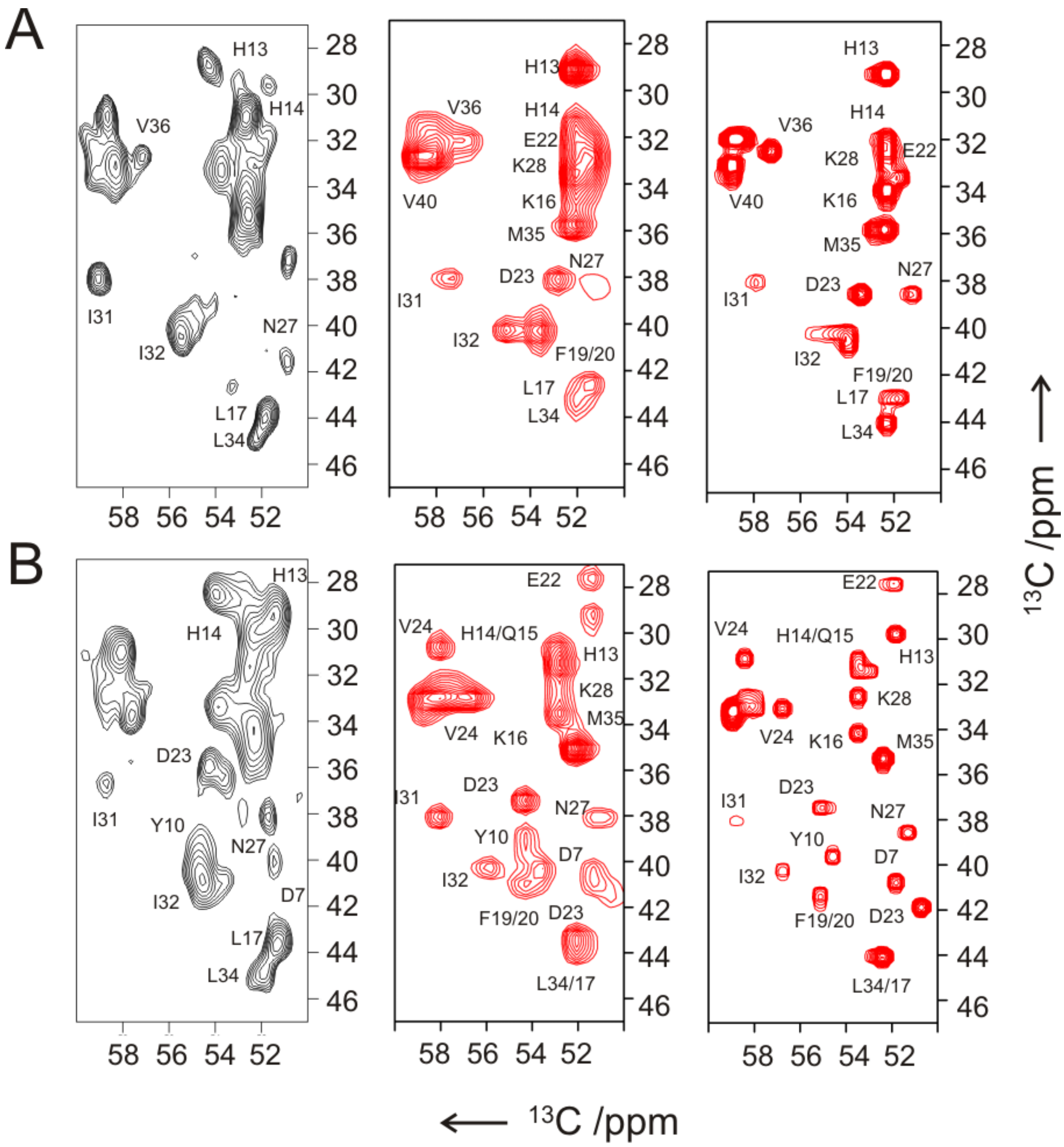

Figure S3: Assignment of ^{13}C-^{13}C DARR SSNMR spectra. A) Spectra showing Cα chemical shifts for 3Q fibrils (black) with corresponding simulated spectra produced using published chemical shift values (red). [2] B) Spectra showing Cα chemical shifts for 2A fibrils (black) with corresponding simulated spectra produced using published chemical shift values (red). [1] The two simulated spectra for each morphology differ only in their line widths (corresponding to T_2 relaxation times of 4 ms (middle) and 10 ms (right).

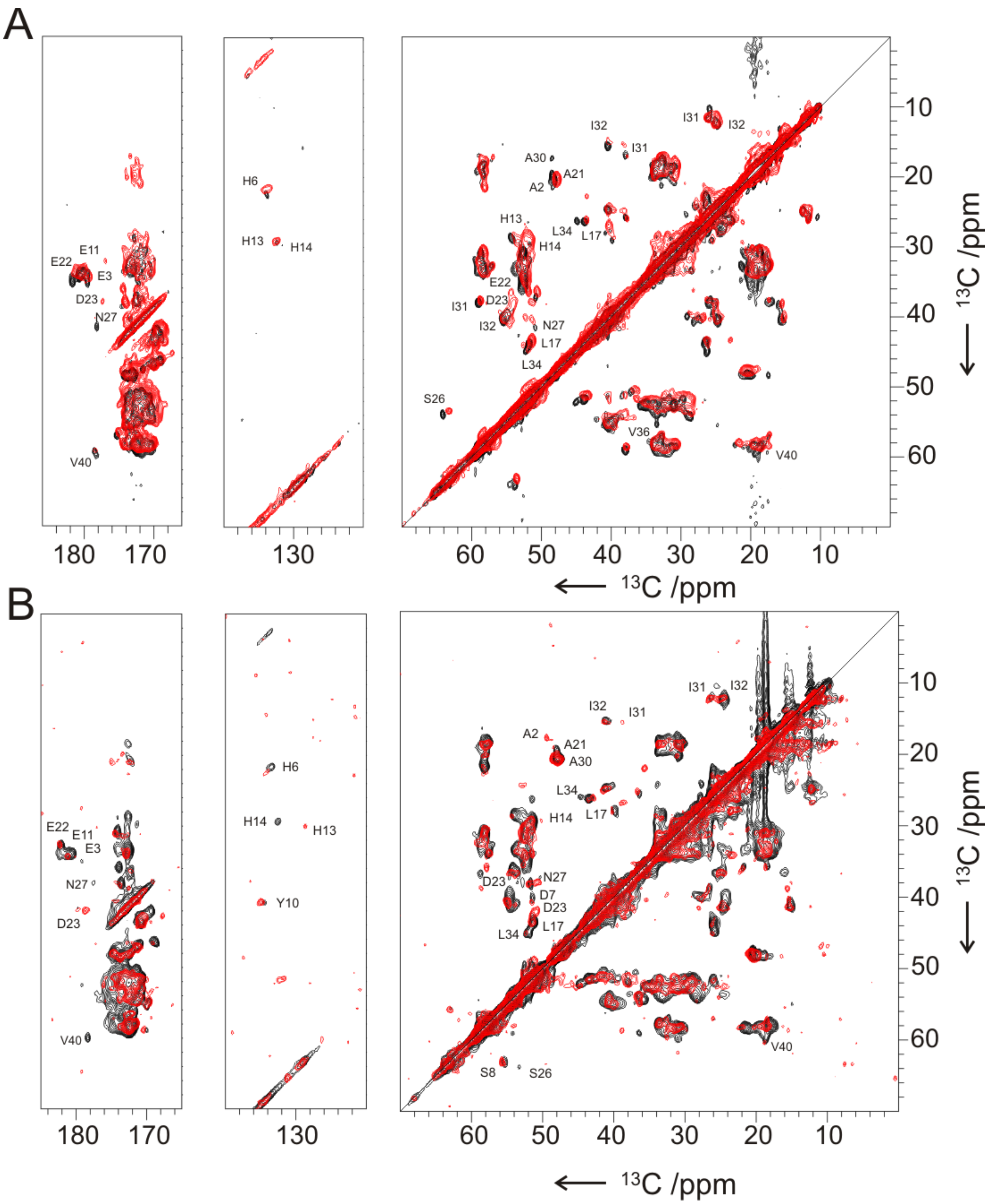

Figure S4: Two-dimensional ^{13}C SSNMR spectra of seeded A$\beta_{1\text{-}40}$ fibrils assembled in the absence (black) or presence of a five-fold mass excess of heparin (red). A) 3Q fibrils. Chemical shift values are summarized in Table S1 and heparin-induced perturbations are shown in Figure 2B (left) of the main text. B) 2A fibrils. Chemical shift values are summarized in Table S2 and heparin-induced perturbations are shown in Figure 2B (right) of the main text.

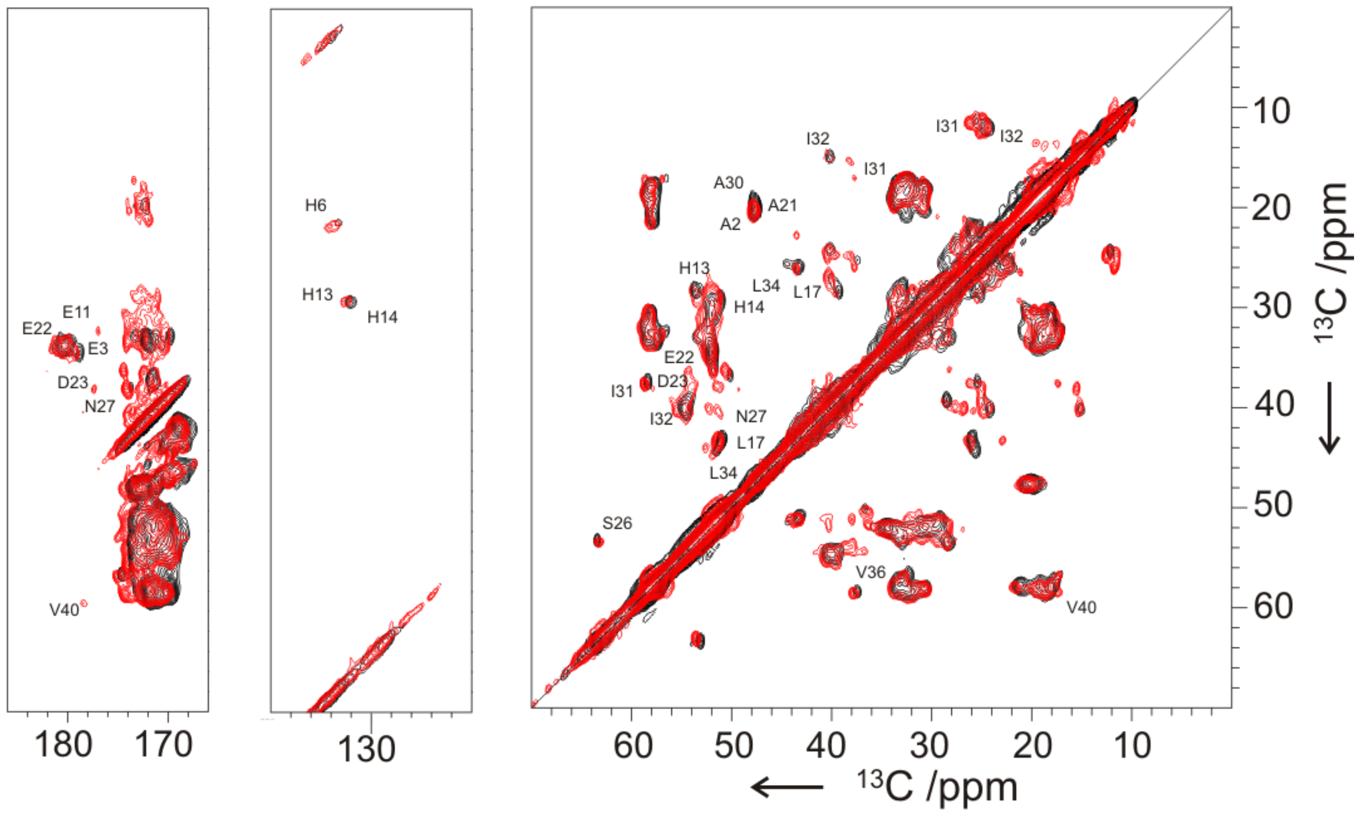

Figure S5: Two-dimensional ^{13}C SSNMR spectra of 3Q A$\beta_{1\text{-}40}$ fibrils formed in the presence of heparin (red) or formed alone with heparin added post-fibril formation (black). Heparin was present in a 5-fold mass excess over the peptide in both cases.

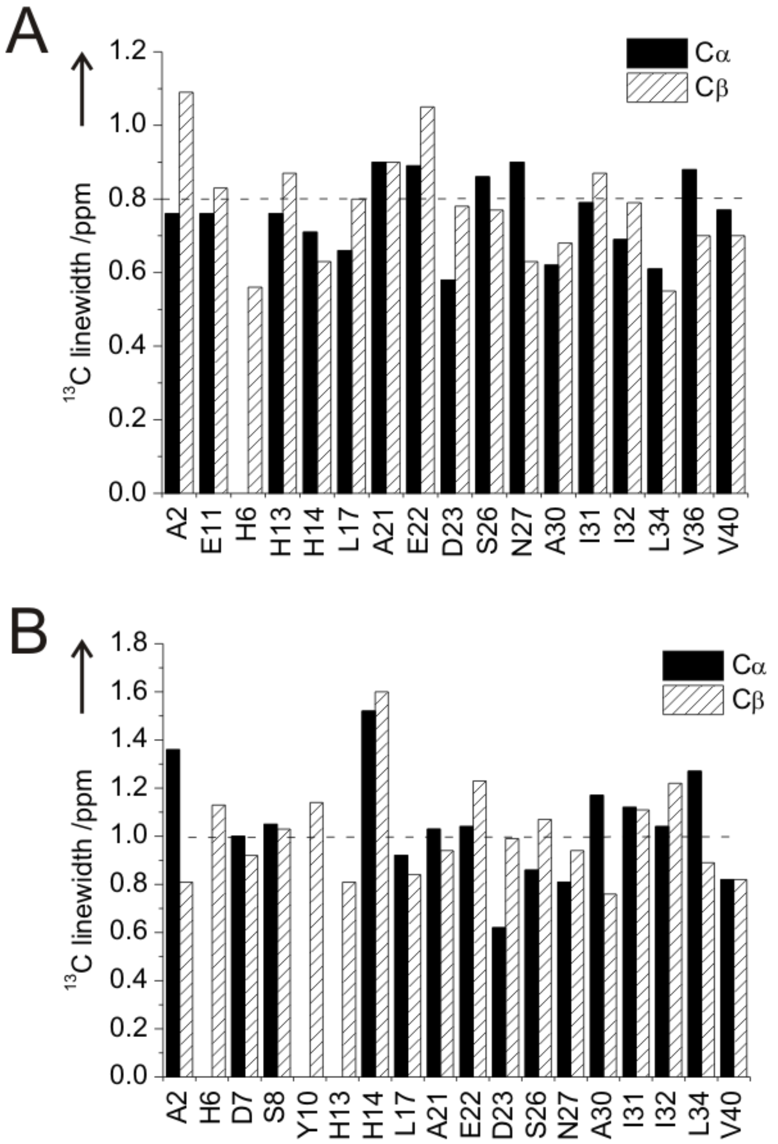

Figure S6: Residue-specific ^{13}C NMR line widths for Cα and Cβ of A$\beta_{1\text{-}40}$ fibrils in the 3Q (A) and 2A (B) morphologies. The average line width at half height is 0.77 ppm for 3Q fibrils and 1.03 ppm for 2A fibrils.

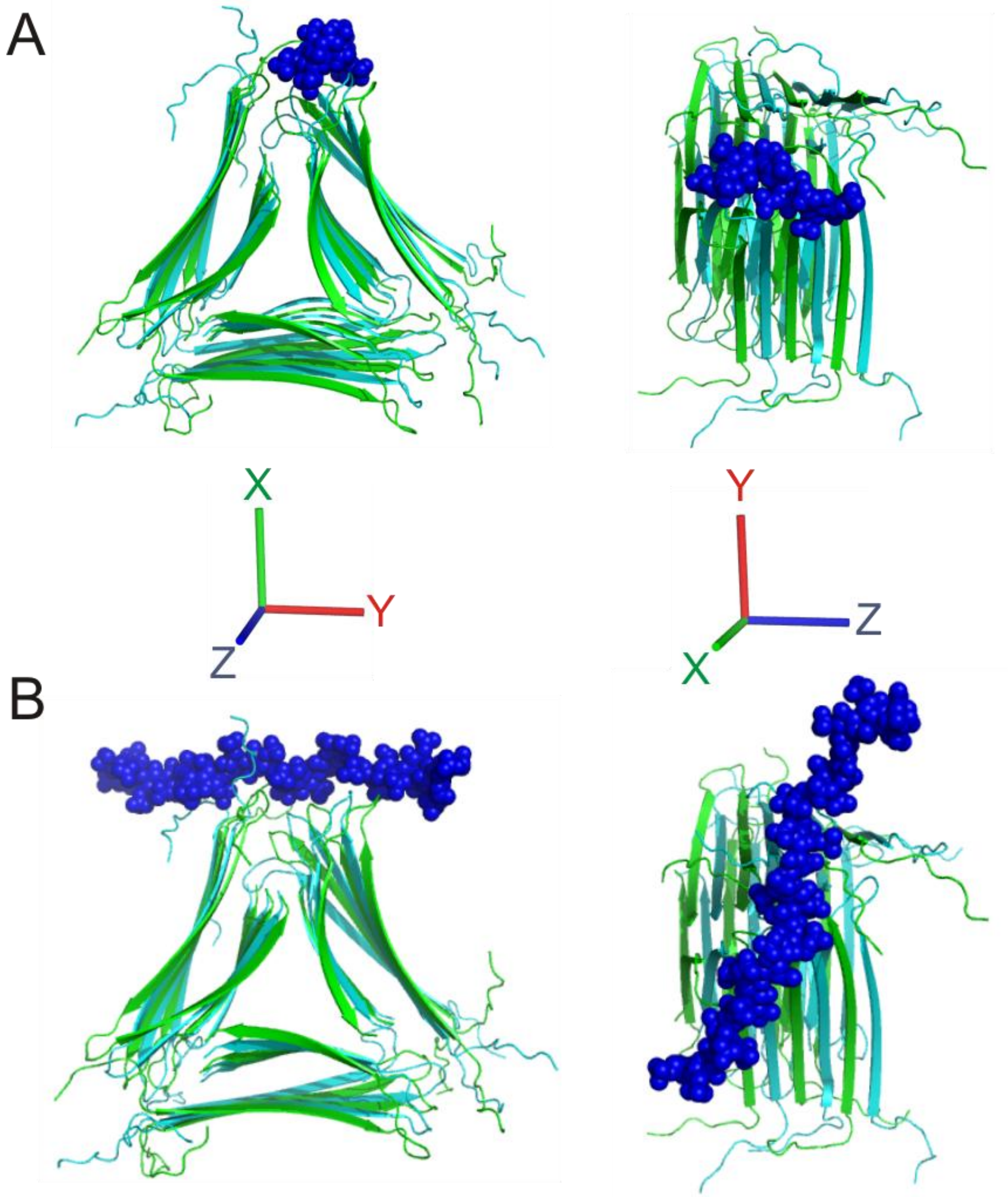

Figure S7: Autodock Vina generated models of heparin bound to 3Q fibrils generated using NMR chemical shifts and spin diffusion data to define search area as described in the main text. A) Heparin arranged along fibril axis *z*. B) Heparin positioned perpendicular to the fibril axis. Heparin (blue spheres), fibril structure (green and cyan). Left-hand image: view along the fibril axis, right-hand image, view perpendicular to the fibril axis. Images generated using PyMOL molecular graphics system, Schrödinger, LLC.

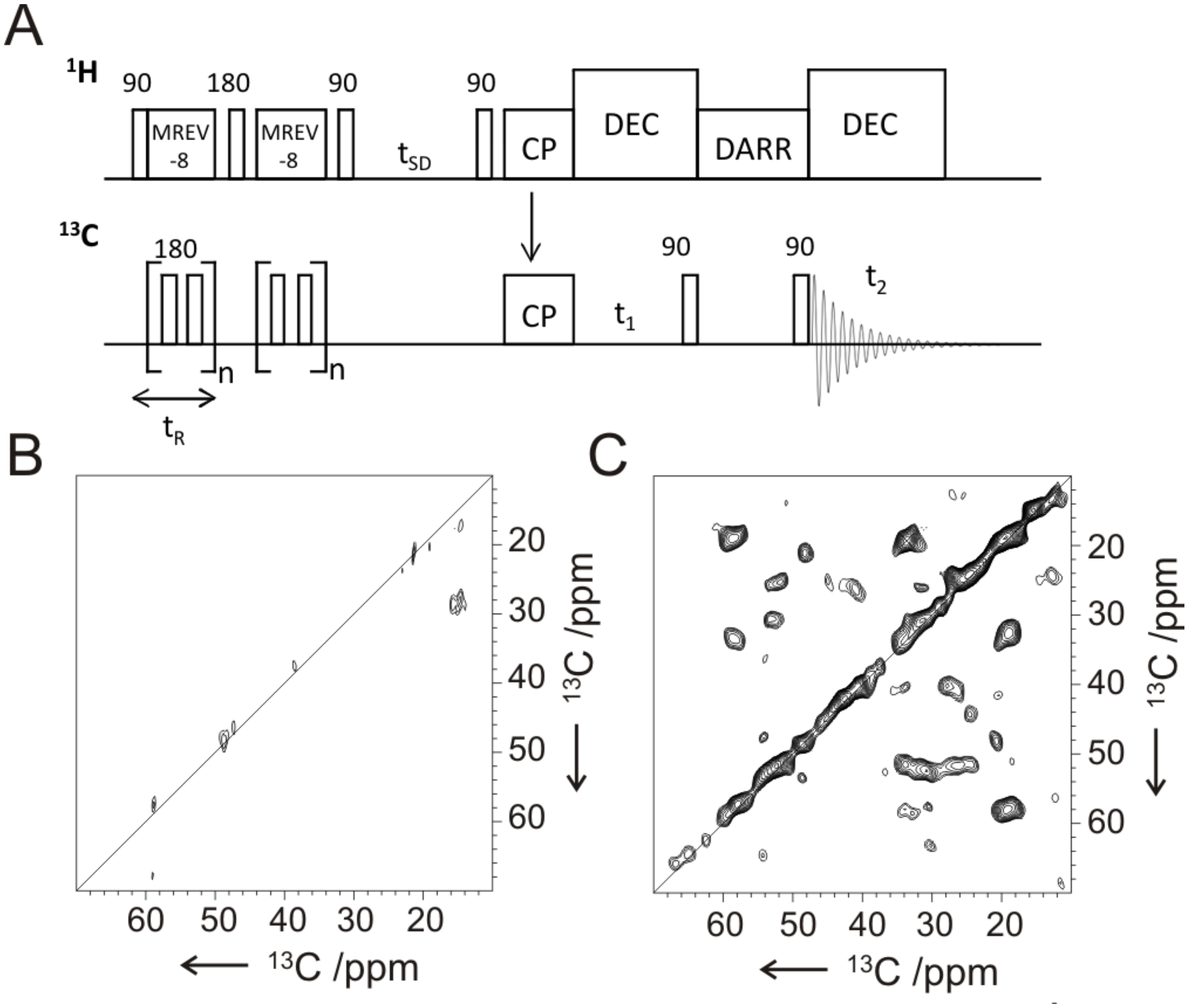

Figure S8: Details of the ^{13}C-detected proton spin diffusion experiment shown in Figure 3C of the main text. A) The pulse sequence. B) Spin diffusion spectrum for A$\beta_{1\text{-}40}$ 3Q plus heparin with t_{SD} set to 5 μs. C) Spin diffusion spectrum with t_{SD} set to 80 μs.

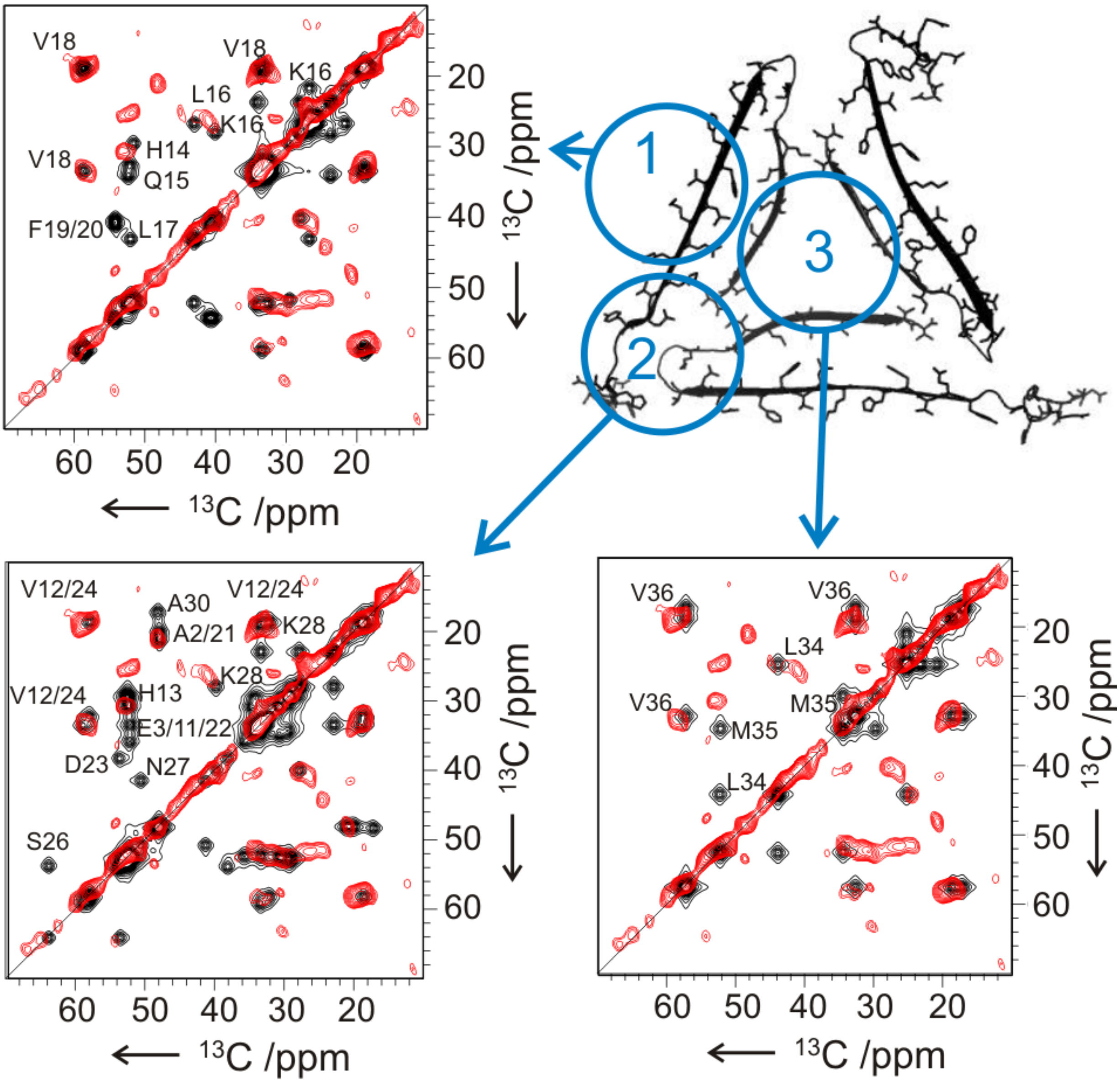

Figure S9: Comparison of the ^{13}C-detected proton spin diffusion spectrum shown in Figure 3C of the main text with simulated spectra for 3Q fibrils with heparin bound to each of three possible regions, 1, 2 and 3, bounded by the circles. In each case the experimental spectrum (red) is superimposed with the simulated spectrum (black). The simulated spectra show contours representing only the spin-systems for the residues confined within the bounded regions. The closest match as judged by qualitative inspection of spectral overlap corresponds to heparin binding within region 2.

Table S1: Summary of ^{13}C chemical shifts for 3Q Aβ_{1-40} fibrils, measured from the SSNMR spectra shown in Figure 2A (left) and Figure S3A. Values in bold are for Aβ_{1-40} alone, with shifts for samples prepared in the presence of heparin shown on the line below. Shifts that are split are indicated by # and the average value is given. Peaks that show a reduction in intensity upon heparin binding are represented by *.

Residue	^{13}C NMR chemical shift (ppm)					
	Cα	Cβ	Cγ	Cδ	Cε	Cζ
A2	**48.2**	**21.4**				
	*	*				
E3			**35.0**	**179.5**		
			34.3	179.2		
R5						**157.8**
						157.3
H6		**22.5**	**133.9**			
		21.7	134.1			
Y10						**154.9**
						154.7
E11			**34.2**	**180.6**		
			33.8	180.7		
H13	**53.0**	**29.3**	**132.4**			
	53.5	29.3	132.4	115.8		
H14	**51.7**	**29.7**	**131.7**			
	51.6	29.1	*			
L17	**51.8**	**43.8**	**26.4**			
	51.5	43.5	26.1			
A21	**48.2**	**20.2**				
	47.8	20.2				
E22	**52.6**	**31.0**	**35.0**	**181.7**		
	52.4	31.6	34.5	182.6		
D23	**54.0**	**38.4**	**177.8**			
	54.0	38.1	177.4			
S26	**53.9**	**64.2**				
	53.4	63.2				
N27	**50.9**	**41.6**	**178.2**			
	51.4	40.3	178.5			
A30	**48.5**	**17.4**				
	49.3	16.9				
I31	**59.1**	**38.0**	**26, 17**	**11.7**		
	58.7	37.6	26.2, 16.3#	11.6#		
I32	**55.5**	**40.4**	**24.7, 15.7**	**12.5**		
	54.8	40.2	25, 15	11.9		
L34	**52.1**	**44.5**	**26.3**			
	52.6	44.0	25.8			
V36	**57.1**	**32.7**	**18.4**			
	57.0	32.7	18.3			
V40	**59.3**		**17.7**			
	59.2		*			

Table S2: Summary of ^{13}C chemical shifts for 2A A$\beta_{1\text{-}40}$ fibrils, measured from the SSNMR spectra shown in Figure 2A (left) and Figure S3B. Values in bold are for A$\beta_{1\text{-}40}$ alone, with shifts for samples prepared in the presence of heparin shown on the line below. Shifts that are split are indicated by # and the average value is given. Peaks that show a reduction in intensity upon heparin binding are represented by *.

Residue	^{13}C NMR chemical shift (ppm)					
	Cα	Cβ	Cγ	Cδ	Cε	Cζ
A2	**49.4**	**17.0**				
	49.5	17.5				
E3			**32.8**	**182.1**		
			32.6	182.3		
R5						**158.4**
						158.2
H6		**21.6**	**133.4**			
		22.0	133.9			
D7	**51.4**	**40.1**				
	51.4	40.1				
S8	**55.7**	**62.9**				
	55.8	63.1				
Y10		**40.8**		**134.5**		**157.2**
		40.7		134.9		157.3
E11			**33.7**	**181.9**		
			*	*		
H13		**29.8**	**128.4**			
		29.9	128.7			
H14	**51.5**	**29.4**	**132.6**			
	51.8	29.5	*			
Q15			**35.0**	**179.2**		
			*	*		
L17	**51.3**	**43.5**	**26.2**			
	51.5	43.5	26.0			
A21	**48.6**	**17.9**				
	*	*				
E22	**52.4**	**24.3**	**33.9**	**180.9**		
	52.4	23.8	34.3	181.2		
D23	**50.1, 53.4**	**41.6, 36.0**	**179.8**			
	50.9, 54.0	42.0, 36.7	178.8			
S26	**53.3**	**63.8**				
	53.9	64.0				
N27	**51.7**	**38.1**	**177.5**			
	52.0	38.3	*			
A30	**47.9**	**20.7**				
	48.1	20.6				
I31	**58.7**	**36.7**	**25.7, 16.5**	**12.3**		
	59.3	37.0	*	12.2		
I32	**54.5**	**40.9**	**24.8, 15.4**	**12.4**		
	55	40.7	25.0, 15.3	12.3		
L34	**51.9**	**44.7**	**26.0**			
	52.3	45.0	*			
V40	**47.6**	**20.7**				
	48.2	20.6				

Methods

Preparation of Aβ peptide

Escherichia coli strain BL21 (DE3) pLysS cells (Merck Chemicals Ltd – Novagen) transformed with the plasmid PetSac expressing $A\beta_{1\text{-}40}$[3] were grown at 37 ˚C in M9 minimal medium containing ampicillin (100 μg/ml), chloramphenicol (25 μg/ml), ^{15}N-ammonium chloride (1 g/l) and ^{13}C-glucose (4 g/l) to obtain uniformly ^{13}C, ^{15}N-labelled peptide, or in LB medium containing antibiotics to obtain unlabelled peptide. This expression system yields $A\beta_{1\text{-}40}$ containing an additional N-terminal methionine residue. Protein expression was induced by the addition of 0.5 mM isopropyl-β-D-thiogalacto-pyranoside at an OD_{600} of 0.5, and the culture was incubated for 18 h at 25 ˚C and harvested by centrifugation (5000 *g* for 15 min at 4 ˚C). Purification was performed as described.[3] Cells were disrupted in 50 mM Tris-HCl, pH 8.5, containing 1 mM ethylenediaminetetraacetic acid and 0.1 mM phenylmethanesulfonyl fluoride, and the extract was centrifuged (18000 *g* for 30 min at 4 ˚C). The pellet was resuspended in 50 mM Tris-HCl, pH 8.5, containing 8 M urea, sonicated, diluted with 3 vols buffer and agitated with Q Sepharose Fast Flow (G E Healthcare). After washing the beads with buffer containing 0 mM NaCl and 25 mM NaCl, peptide-enriched fractions were eluted with 125 mM NaCl. High molecular weight contaminants were removed using 30 kDa MWCO centrifugal filters, and the peptide was concentrated using 3 kDa MWCO centrifugal filters (Vivaspin, Sartorius Stedim). The peptide was purified further by reversed-phase HPLC on a semi-prep C_{18} column (Jupiter, Phenomenex) using a linear gradient of 27-63 % (vol/vol) acetonitrile in 0.1 % (vol/vol) aqueous trifluoroacetic acid, and freeze-dried. Peptide purity was confirmed by Tris-Tricine SDS-PAGE in the presence of reducing agent.[4] The identity and integrity of the purified peptide was verified by electrospray ionization mass spectrometry. Freeze-dried samples were dissolved when required for experiments, and concentrations were estimated from UV absorption in 6 M guanidinium chloride at 280 nm (ε = 1280 M^{-1} cm^{-1}). One litre bacterial culture yielded about two mg of pure uniformly double labelled $A\beta_{1\text{-}40}$.

Sample preparation for solid-state NMR

The unseeded fibril samples were prepared by dissolving the lyophilized peptide (^{13}C-^{15}N or unlabelled $A\beta_{1\text{-}40}$) at a concentration of 0.5 mg/ml in filtered 25 mM sodium phosphate buffer, pH 7.5, 0.02 % sodium azide. Solutions with a volume of 0.5 ml were shaken in 1.5 ml microtubes at 200 rpm and 37 °C for 2 weeks. Additional samples were prepared using buffered solutions of low molecular weight heparin (approx. 5 kDa, Fisher BPE2524) at a concentration of 2.5 mg/ml. Fibrils were pelleted and transferred to 3.2 mm MAS rotors.

The 2A and 3Q seeded fibril samples were prepared by diluting $A\beta_{1\text{-}40}$ fibril seeds[1] to 5 % (v/v) in filtered 25 mM sodium phosphate buffer, pH 7.5, 0.02 % sodium azide, sonicating (2 min, 10 % duty cycle), dissolving the lyophilized peptide at a concentration of 0.9 mg/ml and vortexing. The fibrils were grown quiescently at room temperature for 1 week, with sonication (5 s) after 18 h. Additional samples were prepared using buffered solutions of heparin at a concentration of 4.5 mg/ml.

GAG Binding Assay

Fibril samples were prepared under conditions described above. Fibril samples (0.9 mg/ml) were spun down at 14,000 g for 15 min and supernatant removed, followed by resuspension in varying concentrations of GAG solution as required. Samples were incubated overnight at 25 °C, followed by centrifugation (samples and controls) at 14,000 g for 15 min to pellet fibrils and associated GAG. The level of GAG associated with fibrils was determined by quantifying residual saccharide remaining in solution using heparinase I enzyme (produced in–house). Heparinase I cleaves the glycosidic linkage giving unsaturated uronic acid which can be detected by measuring the absorbance at 232 nm. 125 μl of GAG solution was incubated at 25 °C, 25 μl of heparinase (100-300 units/ml) in buffer (in 20 mM Tris-HCl, 50 mM NaCl, 4 mM $CaCl_2$, 0.01 % bovine serum albumin, pH 7.5) was added, inverted and incubated at 25 °C for 1 hour. The reaction was stopped by addition of 850 μl of 50 mM HCl and levels of uronic acid produced measured at 232 nm. Controls of Aβ fibrils alone and washes of the pellets all showed no GAG presence. GAG binding was tested for varying concentrations (0.1 to 10 fold molar excess) of heparin.

Transmission electron microscopy

A drop of fibril sample was applied to a formvar/carbon-coated copper specimen grid (Agar Scientific Ltd, Stansted, UK), and dried with filter paper before negative staining with 2 % (w/v) aqueous uranyl acetate and drying. Grids were examined in a Phillips CM10 transmission electron microscope operating at 80 kEV.

Linear dichroism (LD)

LD is the differential absorbance of light polarized parallel to an orientation direction and perpendicular to it. The magnitude of signals in flow LD depends on fibre stiffness and length as well as the orientation of transition moments with respect to the flow direction. LD thus enables us to probe orientations of structural motifs such as the β-strands or chromophores such as aromatic residues of the fibrils and to compare the stiffness and length of fibrils of different morphology (such as 2A and 3Q fibrils of $A\beta_{1-40}$), and their ability to interact with other macromolecules such as heparin. To measure LD spectra of different fibril types in the presence or absence of heparin, seeded fibril samples with 2-fold and 3-fold symmetric morphologies were prepared as described above, using filtered 10 mM Na phosphate buffer, pH 7.5. Heparin was dissolved at a concentration of 4.5 mg/ml following fibril growth. Samples containing starch were prepared to control for viscosity effects on fibril alignment. Duplicate samples were prepared to assess reproducibility. Data were recorded using a JASCO J-815 spectropolarimeter adapted for LD measurements. Sample alignment was achieved using a microvolume Couette cell with a rotation speed of 3000 rpm, which was built in-house. Spectra were collected at room temperature from 350-180 nm with a bandwidth of 2 nm, scanning speed of 100 nm/min and data pitch of 0.2 nm. Eight spectra were averaged and a buffer spectrum was subtracted. Samples were diluted 20-fold with buffer to reduce sample absorbance at lower wavelengths.

Solid-state NMR

DARR NMR experiments were performed using a Bruker 850 wide bore spectrometer operating at a static magnetic field of 20 T and using a Bruker 3.2 mm triple resonance probe head in double resonance mode. Samples were maintained at -23°C with a sample rotation frequency of 14 kHz ± 1 Hz. Experiments utilized a ^{1}H 90° excitation pulse length of 2.5 μs, Hartmann-Hahn cross polarization over a 1-ms contact time, 3 μs ^{13}C 90° pulses, SPINAL proton decoupling at 100 kHz and a 1.5 s recycle delay. The proton field was reduced to 14 kHz during the DARR mixing time of 10 ms. Phase-sensitive spectra were obtained using time-proportional phase incrementation with 420 points in the indirect dimension. The spectrum at each t_1 increment was the result of accumulating between 300 and 1024 transients.

^{13}C-detected proton spin diffusion spectra were obtained at a static magnetic field of 9.3 T (400 MHz for ^{1}H) on a Bruker Avance 400 spectrometer at a MAS frequency of 5.1 kHz using a Bruker 4 mm double resonance probe head. The pulse sequence (Figure S8A) was adapted from Kiihne et al.[5] Initially a train of *n* rotor-synchronous REDOR pulses is applied at the ^{13}C frequency whilst simultaneously applying homonuclear decoupling at the proton frequency using MREV-8. Four MREV-8 blocks each of 49 μs duration were applied each rotor period. This step aims to specifically dephase the coherences for protons that are close to ^{13}C. Here, *n* was set to 3, giving a total echo period of 1.2 ms. This echo period was calculated to be sufficient to fully dephase all protons bonded to carbon sites within the ^{13}C fibrils (but only 1 % of carbon-bonded protons in the unlabelled heparin), and also protons separated from ^{13}C by two bonds (i.e., COOH and NH protons of $A\beta_{1\text{-}40}$) and protons within mobile methyl groups. Proton magnetization is then stored longitudinally and a delay t_{SD} allows proton spin diffusion to occur. The range of proton spin diffusion is controlled by the length of t_{SD}. Following ^{1}H-^{13}C magnetization transfer via 100-μs cross-polarization, data were collected in the form of a two-dimensional ^{13}C-^{13}C DARR spectrum with a short (10 ms) mixing time. In this experiment, a short t_{SD} results in little or no signal being detected (Figure S8B) because the residual (non-dephased) proton coherences from heparin have not diffused to $A\beta_{1\text{-}40}$. Signals are observed at longer t_{SD} as a result of proton spin diffusion from heparin to $A\beta_{1\text{-}40}$. At a t_{SD} of 80 μs the recovery of selective signals signifies the $A\beta_{1\text{-}40}$ sites in close contact with heparin (Figure S8C). At t_{SD} times of 200 μs or longer, the selectivity is lost and all signals are recovered. A control spectrum of $A\beta_{1\text{-}40}$ in the absence of heparin shows no signal recovery at a t_{SD} of 80 μs.

Molecular docking procedure

Molecular docking simulation of hexasaccharide and heparin onto $A\beta_{1\text{-}40}$ seeded fibrils was performed using the AutoDock Vina 1.0 package.[6] The co-ordinate pdb files for $A\beta_{1\text{-}40}$ in the 3Q morphology were provided by Dr Robert Tycko, and flexible N-terminal residues were added using Modeller. The co-ordinates were converted into the appropriate format (adding polar hydrogens, removing nonpolar hydrogens and defining rotatable bonds) using AutoDockTools.[7] Residues 1-8 of $A\beta_{1\text{-}40}$ were set as flexible, with the remaining residues held rigid. The rotatable bonds of the glycosidic linkage of heparin were held rigid while all other rotatable bonds were flexible to avoid generation of unrealistic heparin structures.[8] No constraints were imposed on the orientation of

the heparin molecule in the docking procedure. Docking was carried out using a 60 Å cube grid selected in the AutoDock Tools graphical interface. Three-dimensional co-ordinates for this grid were determined to incorporate all of the residues that show significant chemical shift changes in the in the DARR NMR spectra (i.e., with Δ values outside of the region bounded by red lines in Figure 2B). This resulted in a search area that was approximately centred on the apex of the triangular $A\beta_{1-40}$ structure, extending along the length of the fibril axis and into the surrounding space. This was sufficiently large enough to enable the docking process to sample all possible orientations of the heparin molecule on the surface of the $A\beta_{1-40}$ structure. Independent docking simulations were carried out using the default parameters in Autodock Vina 1.0, with each simulation providing a series of clustered outputs. The most energetically favourable conformation was chosen for each simulation. The methods were validated by performing docking calculations for heparin with basic fibroblast growth factor, for which the predicted binding site agreed with the crystal structure (1BFC.pdb) and the calculated free energy of binding (-5.3 kcal/mol) was comparable to the values calculated for heparin binding to $A\beta_{1-40}$.

Simulation of NMR spectra

DARR and ^{13}C-detected proton spin diffusion spectra were simulated using C programs written specifically for that purpose. Simulated DARR spectra were used to assist and validate the assignment of the experimental DARR spectra (Figure S3). Time domain signals were simulated as 512 x 512 matrices and complex Fourier transformation was performed in two dimensions to obtain the frequency domain spectra. The ^{13}C resonance frequencies used in the simulations were taken directly from the chemical shift values reported in Refs 1 and 2, which omitted residues D1, E3, F4, R5, H6, E11 and V40 for the 2A morphology and D1, E3, F4, R5, H6, D7 and S8 for the 3Q morphology. Most of these residues are situated in the unstructured N-terminal region. For the simulated DARR spectra, only short-range couplings between directly bonded spins were considered, and long-range couplings were neglected to simplify the simulated spectra. Each FID in the t_2 dimension was modulated therefore by no more than three frequencies in t_1 (i.e., depending on the number of bonded carbon atoms), which restricted the number of cross-peaks in the simulated spectra. In the experimental DARR spectra, cross-peaks correlating long range couplings were not observed because of the short mixing time (10 ms) used. Comparison of the simulated and experimental DARR spectra revealed a good agreement in the number and frequencies of the cross-peaks, as illustrated by the representative regions in Figure S3. The simulations did not take into consideration the magnitude of the dipolar couplings, dipolar truncation or spin-diffusion effects, which influence the relative intensities of the cross-peaks. Consequently all simulated cross-peaks and diagonal peaks shown have equal intensities.

The simulated spin-diffusion spectra were obtained with the same procedure used for the DARR spectra, but restricting the frequencies to only those residues within the regions 1, 2 or 3 in Figure S9. These regions were selected as follows. First, the size of the circle in region 2 was adjusted to encompass the cluster of residues for which significant chemical shift perturbations were observed in the DARR spectra and are also situated within a 15 Å radius of the predicted interaction interface shown in Figure S7. This radius is estimated to be the upper limit of the spin-diffusion range under the experimental conditions used. Circles of the same size were then used

to encompass residues on the outer and inner faces of the fibril structure to ensure that all residues were covered by the three regions. Spectra were then simulated from the resonance frequencies of the residues in each of the three regions and compared qualitatively with the experimental spectrum.

Supporting References

[1] A. T. Petkova, R. D. Leapman, Z. Guo, W.-M. Yau, M. P. Mattson, R. Tycko, *Science* **2005**, *307*, 262-265.

[2] A. K. Paravastu, R. D. Leapman, W.-M. Yau, R. Tycko, *Proc. Natl. Acad. Sci. USA* **2008**, *105*, 18349-18354.

[3] D. M. Walsh, E. Thulin, A. M. Minogue, N. Gustavsson, E. Pang, D. B. Teplow, S. Linse, *FEBS J.* **2009**, *276*, 1266-1281.

[4] H. Schägger, G. von Jagow, *Anal. Biochem.* **1987**, *166*, 368-379.

[5] S. R. Kiihne, A. F. L. Creemers, W. J. de Grip, P. H. M. Bovee-Geurts, J. Lugtenburg, H. J. M. de Groot, *J. Am. Chem. Soc.* **2005**, *127*, 5734-5735.

[6] O. Trott, A. J. Olson, *J. Comput. Chem.* **2010**, *31*, 455-461.

[7] M. F. Sanner, *J. Mol. Graphics Model.* **1999**, *17*, 57-61.

[8] T. Takaoka, K. Mori, N. Okimoto, S. Neya, T. Hoshino, *J. Chem. Theory Comput.* **2007**, *3*, 2347-2356.
